# Social robots in cognitive and speech rehabilitation for children with cerebral palsy: a scoping review

**DOI:** 10.1186/s12984-025-01852-0

**Published:** 2025-12-19

**Authors:** Aray Zhaisanbek, Saule Karibzhanova, Ihteshamul Hayat, Amina Abdikalyk, Amna Riaz Khawaja, Damira Mussina, Sourav Mukhopadhyay, Prashant Kumar Jamwal

**Affiliations:** 1 Department of Robotics and Mechatronics, School of Engineering and Digital Science, Nazarbayev University, Astana, Kazakhstan; 2 School of Medicine, Nazarbayev University, Astana, Kazakhstan; 3 Department of Electrical and Computer Engineering, School of Engineering and Digital Sciences, Nazarbayev University, Astana, Kazakhstan; 4 Department of Computer Science, School of Engineering and Digital Sciences, Nazarbayev University, Astana, Kazakhstan; 5 Department of Physical Therapy, Athletic Training, and Rehabilitation Science, School of Health Professions, University of Kansas Medical Center, Kansas, KS USA; 6 Graduate School of Education, Nazarbayev University, Astana, Kazakhstan; 7 Nazarbayev University Research Administration (NURA) Private Institution, Astana, Kazakhstan

**Keywords:** Robotics, Socially assistive robots, Cerebral palsy/rehabilitation, Pediatrics, Cognition/rehabilitation, Speech therapy, Communication disorders/therapy, Human–robot interaction, Assistive technology, Patient engagement

## Abstract

**Background:**

Children with cerebral palsy (CP) often experience cognitive, speech, and communication challenges that limit participation and daily functioning. Socially assistive robots (SARs) have emerged as a promising technology to support these domains, yet evidence remains fragmented across different settings, robot types, and intervention approaches. This scoping review aimed to map current research on SAR-based cognitive, speech, and communication rehabilitation for children with CP and to identify trends, gaps, and opportunities for future work.

**Methods:**

The review followed the PRISMA-ScR framework. Six databases (PubMed, Scopus, Web of Science, IEEE Xplore, ScienceDirect, and EBSCOhost) were searched for articles published between 2015 and 2025. Eligible studies involved children aged ≤ 18 years with a confirmed diagnosis of CP and used a social robot with interactive or socially oriented features as part of cognitive, speech, communication, or engagement-focused rehabilitation. Screening and data charting were performed by four reviewers. Extracted data included participant characteristics, robot type, intervention setting, rehabilitation domain, outcome measures, and key findings. Results were synthesized descriptively and thematically.

**Results:**

Twenty studies met inclusion criteria. SAR interventions were delivered in clinical, educational, or home environments and commonly targeted speech production, vocabulary, symbolic play, attention, and social engagement. Across studies, SARs improved motivation, communication attempts, engagement duration, and participation. Humanoid and animaloid robots were both effective as adjunctive tools, with high acceptance among children, parents, and therapists. However, evidence is limited by small sample sizes, short intervention durations, heterogeneous outcome measures, and limited inclusion of children with severe motor or communication impairments. Few studies evaluated long-term effects or standardized rehabilitation protocols.

**Conclusions:**

Current evidence suggests that SARs are feasible and engaging tools that can enhance cognitive, speech, and communication rehabilitation for children with CP. To advance clinical integration, future research should include longer and larger trials, standardized outcome measures, personalization strategies, and assessments across functional classifications. Strengthening methodological rigor will be essential for translating SARs from prototypes into validated rehabilitation technologies.

*Trial registration* Not applicable; this scoping review did not involve human participants in an intervention.

## Introduction

### Background

Cerebral palsy (CP) is the most prevalent neurodevelopmental disorder in children worldwide, impacting over 17 million individuals across the globe [1, 2]. For clarity, the abbreviations used in this review are summarized in Table 6. CP is a long-term neurophysiological disorder characterized by abnormal tone, posture, and movement. CP is a group of permanent, non-progressive disorders that affect the development of movement and posture, causing activity limitation. This is attributed to non-progressive damage that occurs in the developing brain of a fetus or infant [3]. The classification of CP could be based on the predominant motor syndrome: spastic hemiplegia, spastic diplegia, spastic quadriplegia, and dyskinetic [4, 5].

Children with CP are most frequently accompanied by epilepsy, secondary musculoskeletal issues, and disruptions in sensation, perception, cognition, communication, and behavior [2, 6–8]. These disorders arise from disturbances in early brain development before the nervous system has fully matured, typically before the age of five [8, 9]. Well-established risk factors include maternal infection, multiple gestation, prematurity, and low birth weight [2, 8]. In many cases, the initial injury to the brain occurs during early fetal development, which could be attributed to intracerebral hemorrhages and periventricular leukomalacia [2, 8, 10]. Although CP itself is a non-progressive disorder, secondary complications—particularly musculoskeletal deformities and epilepsy—often progress over time if not adequately managed [2, 8].

In recent years, there has been a gradual shift in the rehabilitation of children with CP; the focus is more on technological integration, such as the use of social assistive robots (SARs). SARs have been positively linked with improving cognition, communication, and social skills [4, 11]. A study with an NAO robot revealed that motivation, occurring in relation to the robot, contributed to persistence in physical therapy and achieving SMART goals in boys with a dyskinetic type of CP [12]. In virtual environments with humanoid child representations, non-verbal social interaction capabilities have been enhanced—they have been repeating socially important gestures longer and more actively, which increases therapeutic time and the efficiency of exercises [13]. Additionally, a game study with Lego robots demonstrated that a child’s dependence on adults in gaming decreases, while speech activity (for example, responding to yes/no questions) develops independently, leading to the growth of communicative autonomy [14]. Similarly, the experiment with LekBot robots showed that during a joint game activity, the children exhibited mutual involvement, positive emotions, and elements of joint initiative—signs of symmetrical and joyful social interaction [15].

### Cerebral palsy type/classification or clinical presentation

Children with CP are often faced with cognitive, communication, and social skills challenges, which vary depending on the type and severity of the disorder. About 30% of children with CP, regardless of CP subtype, have intellectual impairments [16], which are frequently accompanied by severe visuo-perceptual/visuo-constructive issues. In children with typical cognitive levels, CP is linked to a high prevalence of certain learning difficulties [17], reduced processing speed [18], and problems with working memory, sustained attention, and inhibitory control [19]. Also, people with CP have a higher chance of dropping out of school and the workforce [20], and they exhibit more signs of conduct disorders, anxiety, and attention-deficit/hyperactivity disorder (ADHD) [6]. Social and emotional issues manifest in establishing contacts with peers, expressing negative emotions, and research activity, especially in young children [7]. Communicative disorders are regularly linked with dysarthria, impairment of articulation, and limited motor skills of speech organs, which makes it difficult for verbal communication [21]. Diagnosis of these impairments is conducted with the use of various scales and classifications, such as GMFCS for assessment of motor function, the scale MACS for assessment of manual activity, and the scale CFCS for assessment of communicative abilities [22]. However, CP follow-up programs mainly concentrate on physical functioning, and cognitive ability is frequently assumed rather than evaluated [9]. It is important to note that children with more severe forms of CP, as a rule, are less likely to undergo cognitive assessment, despite the high needs for an individualized approach [23].

### Pathophysiology and its broader impact

The underlying pathophysiology of CP is linked to early brain injury affecting motor and sensory pathways, including the corticospinal tract, basal ganglia, and cerebellum [8]. Key pathogenetic factors include hypoxia–ischemia, inflammation, oxidative stress, and disrupted neuronal or oligodendrocyte maturation. These processes often lead to white matter injury, necrosis, and impaired myelination, which interfere with both motor and sensory signal transmission [10].

Importantly, these disturbances extend beyond motor pathways. Thalamocortical and other sensory projections are also impaired, limiting the processing of proprioceptive, tactile, and visual information [8, 24]. As a result, children with CP often struggle with cognition, memory, language, and social communication. Deficits in sensory integration and altered brain connectivity further compromise attention, speech development, and social cognition. Impaired integration of sensory information and altered brain connectivity also limit attention, speech skills, and social cognition, which aggravate the communication and social participation of children [8].

At the same time, the developing brain retains a remarkable capacity for neuroplasticity, which provides opportunities for targeted rehabilitation. This potential has sparked growing interest in technology-driven approaches, including the use of socially assistive robots (SARs), as structured and motivating tools for therapy [25].

### Beyond motor rehabilitation: social and cognitive needs

Children with CP face lifelong challenges that extend beyond motor control, encompassing cognitive, communicative, and social functioning [26]. Therefore, rehabilitation of these children extends beyond physical therapy and often requires approaches that foster engagement, motivation, and social interaction. In this context, technological innovations such as social robots have gained attention as promising tools to support cognitive therapy. SARs are designed to interact with humans through verbal, non-verbal, and affective communication, often adopting humanoid or animal-like forms [27, 28]. Their defining feature lies in their capacity for meaningful interpersonal engagement, making them well-suited for pediatric rehabilitation [27, 28].

### Evidence for social assistive robotics for rehabilitation

Social robots have immense potential for applications in therapy, education, and mental health support. They have been well-integrated into a wide range of fields, including healthcare, rehabilitation, and caregiving. Evidence indicates that interventions with social robots can enhance social connection, communication, and emotional well-being in both children and adults, while also reducing anxiety, depression, and fear [29]. Similar findings have been reported regarding their role in mitigating social anxiety [30], and further studies show positive effects on stress regulation and emotional affect in children [31].

A promising subset of this field is the development of SARs. In the context of cognitive and speech rehabilitation, SARs offer consistent, engaging, and fatigue-free support qualities that are increasingly valuable considering the rising demands on healthcare professionals and the global shortage of skilled therapists. It is that SARs offer the advantage of delivering highly repeatable interventions without the limitations associated with human fatigue or emotional strain [32]. Evidence from related clinical populations, such as patients with mild cognitive impairment, also suggests that social robots can support cognitive stimulation (e.g., memory and executive functions) and act as mediators in social interaction, highlighting their broader potential for cognitive and social rehabilitation across diverse groups [33]. Emerging work in affective computing further shows that social robots equipped with emotional communication abilities—through facial expressions, gestures, and adaptive responses—can inspire curiosity, foster engagement, and act as companion-like supports in therapy, underscoring the potential of SARs to address not only cognitive but also socio-emotional needs of children with disabilities [34].

### Gaps in current literature

Despite growing interest in SARs for children with CP, the existing literature remains fragmented, with most systematic reviews and meta-analyses narrowly focused on physical rehabilitation outcomes—particularly gait and motor function [35–37]. While studies consistently highlight high engagement [38, 39] and the motivational value of SARs like the NAO robot [40, 41], they are often limited in scope, sample size, and methodological rigor. Moreover, most prior reviews emphasize short-term feasibility trials, lack standardized outcome measures, and rarely explore cognitive, communicative, or speech-related rehabilitation in depth [32–35, 38, 39, 42, 43].

A scoping review was chosen because it enables the broad mapping of heterogeneous evidence, the identification of key concepts, and the identification of research gaps in socially assistive robotics for children with cerebral palsy [26].

Additionally, previous reviews have typically underrepresented the role of therapists and caregivers, offered limited insights into the longitudinal effects or deployment in clinical settings, and have not adequately addressed technological diversity beyond a few dominant platforms [40, 41]. There is also a lack of synthesis concerning personalization strategies, multimodal interaction methods, or the integration of SARs into hybrid cognitive-physical rehabilitation models.

### Research question

By conducting this scoping review, we aim to systematically chart the current landscape of socially assistive robot (SAR) use for cognitive, speech, and communication rehabilitation in children with cerebral palsy. The review seeks to identify the types of SARs applied in therapeutic contexts, summarize reported outcomes and interaction strategies, and map methodological characteristics, limitations, and evidence gaps across existing studies. Accordingly, this scoping review addresses the following research questions:

- What types of socially assistive robots have been used in cognitive, speech, and communication interventions for children with cerebral palsy?
- What therapeutic outcomes, interaction strategies, and engagement measures have been reported across existing studies?
- What methodological characteristics, limitations, and evidence gaps are present in current research on SAR-based rehabilitation for children with cerebral palsy?

## Methodology

This scoping review was conducted in accordance with the Preferred Reporting Items for Systematic Reviews and Meta-Analyses extension for Scoping Reviews (PRISMA-ScR). The review aimed to map the existing literature on the use of social robots for cognitive, speech, and communication rehabilitation in children with CP.

Clinical trial number: not applicable.

Protocol registration: not applicable.

### Search strategy

A comprehensive literature search was conducted across six electronic databases: PubMed, Scopus, IEEE Xplore, EBSCOhost, ScienceDirect, and Web of Science. The search strategy was tailored to the specific indexing and functionality of each database, employing a combination of controlled vocabulary (such as MeSH terms) and free-text keywords related to social robots, cerebral palsy, children, and rehabilitation. Boolean operators (AND, OR, NOT) and database-specific filters were utilized to refine the results and enhance relevance.

Used search strings can be found in Table 1. Search filters applied across all databases included a publication date range restricted to the past 10 years (2015–2025), a target population limited to children aged 0–18 years, and an English language requirement, as well as access to either open-access or free full-text documents. Eligible document types included peer-reviewed articles and conference papers. Studies were excluded if they focused on gait, exoskeletons, or involved solely physical or motor rehabilitation without a social or communicative component. All records were exported in RIS format and managed in EndNote for duplicate removal. The PRISMA-ScR guidelines were followed. The PRISMA-ScR checklist is available as Supplementary File.

**Table 1:** Search strategy

Database	Keywords	Hits
PubMed	(social robot) AND (rehabilitation) AND (cerebral palsy) AND (children) NOT (gait OR exoskeleton)	107
EBSCO	((robots) AND (cerebral palsy or cp) AND (children or kids or youth or child) AND (rehabilitation) NOT (covid-19 OR farming OR socioeconomics OR gait OR exoskeleton OR ankle OR upper limb)	77
Scopus	(social robot) AND (rehabilitation) AND (cerebral palsy) AND (children) NOT (gait OR exoskeleton)	41
IEEE Xplore	("social robot" OR "assistive robot" OR "rehabilitation robot" OR "humanoid robot") AND ("cerebral palsy" OR CP)	412
Science direct	(Social robot OR assistive robot) AND (rehabilitation OR therapy) AND (cerebral palsy children) AND (speech) AND NOT (exoskeleton OR walking)	258
Web of science	TS = ("social robot" OR "assistive robot" OR "rehabilitation robot" OR "humanoid robot") AND TS = ("cerebral palsy" OR "CP") AND TS = (child* OR children OR youth) AND TS = (rehabilitation OR therapy OR speech OR cognition) NOT TS = ( "gait" OR "exoskeleton" OR "upper limb" OR "lower limb")	36

### Screening

**Fig. 1:**
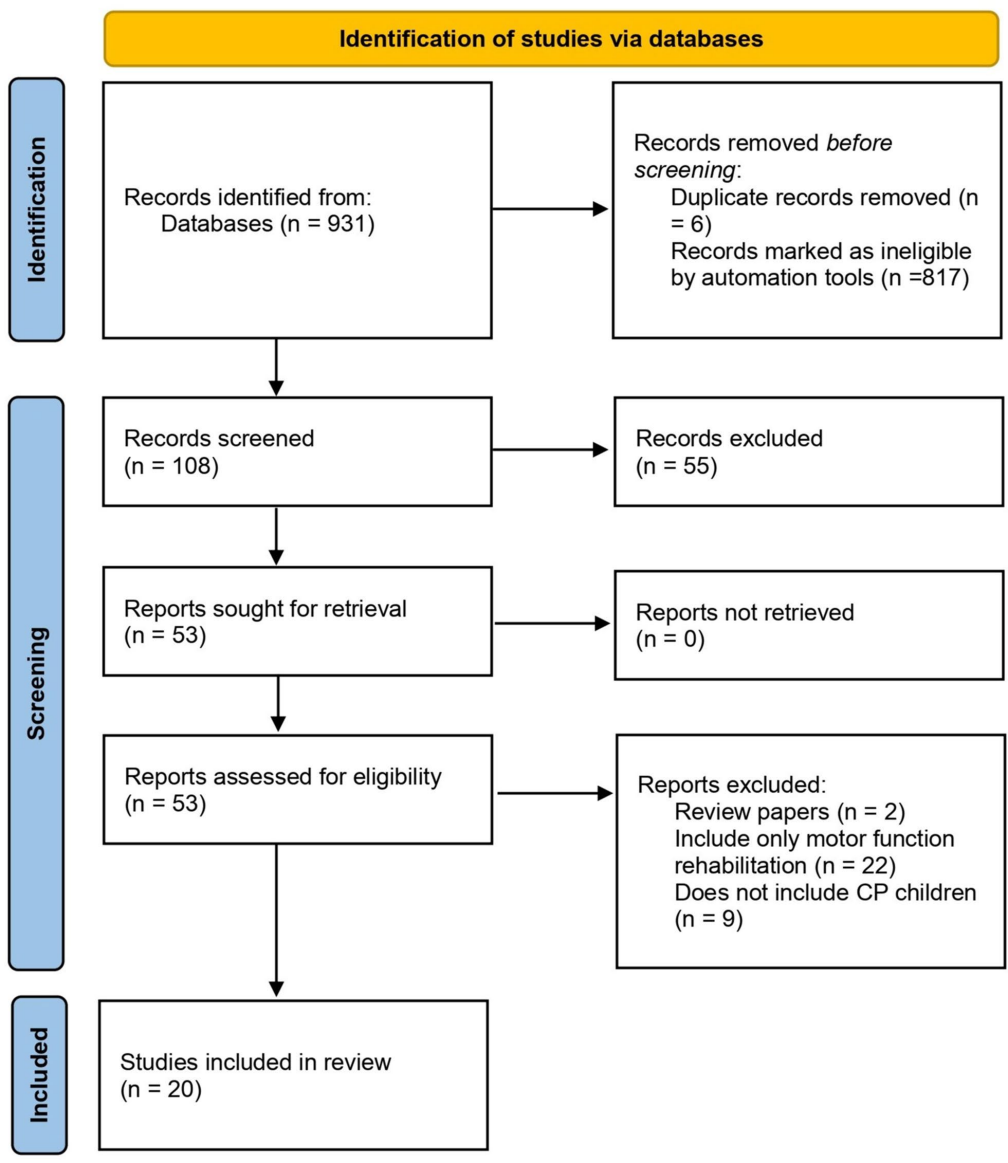
PRISMA flowchart reflecting the process of article selection

All records retrieved through the database searches were manually checked, and duplicates were identified and removed. Title and abstract screening were carried out independently by four reviewers according to predefined inclusion and exclusion criteria. Because each reviewer screened a distinct dataset, formal interrater reliability (e.g., Cohen’s kappa) could not be calculated. Any uncertainties or borderline cases were discussed collectively, and discrepancies were resolved by consensus between reviewers. After abstract screening, full-text versions of potentially eligible articles were retrieved and reviewed for final inclusion. After full-text revision, 20 articles were identified as eligible for inclusion. The screening and selection process is illustrated in the PRISMA flow diagram (see Fig. 1). Studies were selected according to predefined exclusion and inclusion criteria to ensure relevance and quality. Inclusion and exclusion criteria can be found in Table 2.

**Table 2:** Inclusion and exclusion criteria

Category	Inclusion criteria	Exclusion criteria
Population	Children (≤ 18 years) diagnosed with CP	Adults or mixed-age samples without separate analysis for children
Condition/diagnosis	Participants specifically diagnosed with CP	Studies focusing on other disabilities (e.g., autism, ADHD) without a distinct CP subgroup
Intervention type	Use of social robots displaying social features (e.g., verbal interactions, gestures, emotional expression)	Robots used exclusively for physical support or assistance without social interaction
Purpose of intervention	Rehabilitation aimed at cognitive, speech, or communication improvement	Intervention unrelated to cognition, communication, emotion, or behavior
Outcome measures	Studies reporting outcomes such as social engagement, communication skills, cognitive development, or behavioral responses	Studies not reporting the above outcome types
Study design	Empirical studies with real-world testing or user interaction (e.g., feasibility studies, trials, case studies)	Reviews, editorials, opinion pieces, or other non-empirical works
Source	Peer-reviewed journals	Grey literature, theses, reports
Language and availability	Full text available in English	Non-English publications or unavailable full texts
Duplicates		Duplicate publications; only the most comprehensive version retained

### Data extraction and synthesis

A structured data extraction form was used to collect relevant information from each included study. Extracted data included bibliographic details (author, year, journal), study location, design and sample size, type and technical features of the robot, targeted rehabilitation domain (speech, cognitive, communication), duration and setting of the intervention, outcome measures and key results, user feedback (from children, caregivers, or therapists), and any reported limitations. Data extraction was performed independently by four reviewers and cross-checked for consistency and accuracy, with disagreements resolved by consensus.

### Eligibility

The extracted data were synthesized narratively and thematically. Studies were grouped based on the type of rehabilitation addressed, the features and roles of the robots, the setting and mode of intervention delivery, and the outcomes observed.

## Results

### Study characteristics

A total of 20 studies were included in this review, which was published between 2015 and 2025. The research was geographically diverse, and it included contributors from 13 countries. Such as USA (n = 3), Malaysia (n = 3), Australia (n = 2), Spain (n = 3), Serbia (n = 2), the United Kingdom (n = 1), UAE (n = 1), Colombia (n = 1), Brazil (n = 1), Ecuador (n = 1), India (n = 1), Canada (n = 1), China (n = 2), Italy (n = 1), and Japan (n = 1). The majority of studies originated from Western and European countries, though representation from Latin America and Asia is also strong. Some countries collaborated on common research. For example, China and Serbia, or India and Canada.

Across the 20 included studies, a wide range of study-design types was reported, and several articles used more than one design. Feasibility studies appeared in 4 studies (20%), and usability studies in 1 study (5%). Pilot designs were common overall, including general pilot studies in 3 studies (15%), pilot experimental studies in 2 studies (10%), and pilot observational studies in 1 study (5%). Design-oriented contributions included design and validation studies (1 study, 5%), system design/performance evaluations (1 study, 5%), prototype development and implementation studies (1 study, 5%), and technical validation studies (1 study, 5%). Additional designs included a single-case experimental design (1 study, 5%), a longitudinal intervention study (1 study, 5%), an experimental user study (1 study, 5%), and an observational study (1 study, 5%). Qualitative and conceptual approaches were represented by conceptual/methodological papers (1 study, 5%) and qualitative or reflective observational studies (2 studies, 10%). Case-based evidence included case studies (1 study, 5%) and a case-based pilot intervention (1 study, 5%). One study employed a randomized controlled trial design (1 study, 5%). This distribution reflects substantial methodological heterogeneity across the literature, with many studies adopting multiple design types to address both technical development and preliminary clinical evaluation. Notably, several studies incorporated multidisciplinary approaches, involving collaboration between engineers, clinicians, and therapists, with some employing gamified or multimodal interaction frameworks to enhance engagement.

Overall, the literature demonstrates an emerging but fragmented evidence base, with considerable variability in methodological rigor, duration of interventions, and evaluation metrics.

### Targeted outcomes

The reviewed studies reported six main outcome domains for robot-assisted rehabilitation in children with cerebral palsy: speech production, language comprehension, attention/memory, social communication, symbolic play, and motor function with social interaction.

#### Speech production

Speech production and articulation were a central focus, with robots supporting phonological practice and speech recognition. One study developed a Japanese-language ASR dataset of 429 sentences to improve recognition of dysarthric speech [44], while others demonstrated improvements in phonological skills and morphosyntax through structured, game-based interventions [45].

#### Language comprehension and expression

Language comprehension and expressions were also targeted. A speech-therapy assistant delivered semantic and morphosyntactic exercises with automated logging [45], while the MARKO humanoid enabled children to follow instructions and sustain short dialogues [46]. Integrated HCI systems framed language training as one of two core modules, supporting both receptive and expressive skills [47].

#### Attention and memory

Attention and memory were also frequently embedded into therapy design. Gamified systems like REHAB-PAL used scoring, feedback, and adaptive questioning to maintain focus [48]. Memory-specific games such as the NAOTherapist “Simon” task, trained working memory and attention while supporting motor practice [49]. Other interventions explicitly listed cognition, attention, and memory as primary outcomes [50], or adapted robot feedback to engagement dynamics [51].

#### Social communication and pragmatics

In addition to this, social communication and pragmatics were enhanced through both verbal and non-verbal training. Robots supported gestures (shrugging, negating, showing surprise) [13], turn-taking, joint attention, and conversational reciprocity [52, 53]. Home-based systems reinforced pragmatic skills through praise and clarifying questions [48, 54], while role-reversal designs encouraged empathy and socially guided exchanges [55].

#### Symbolic play and interaction skills

Symbolic play and interaction skills were fostered through puzzle-solving, storytelling, and daily-life gesture games [56]. A multiple-baseline study showed that robot-mediated free play improved playfulness in children with severe motor limitations, with gains persisting at follow-up [57]. Gamified platforms also promoted imaginative play and therapy adherence [49].

#### Motor rehabilitation with social interaction

**Fig. 2:**
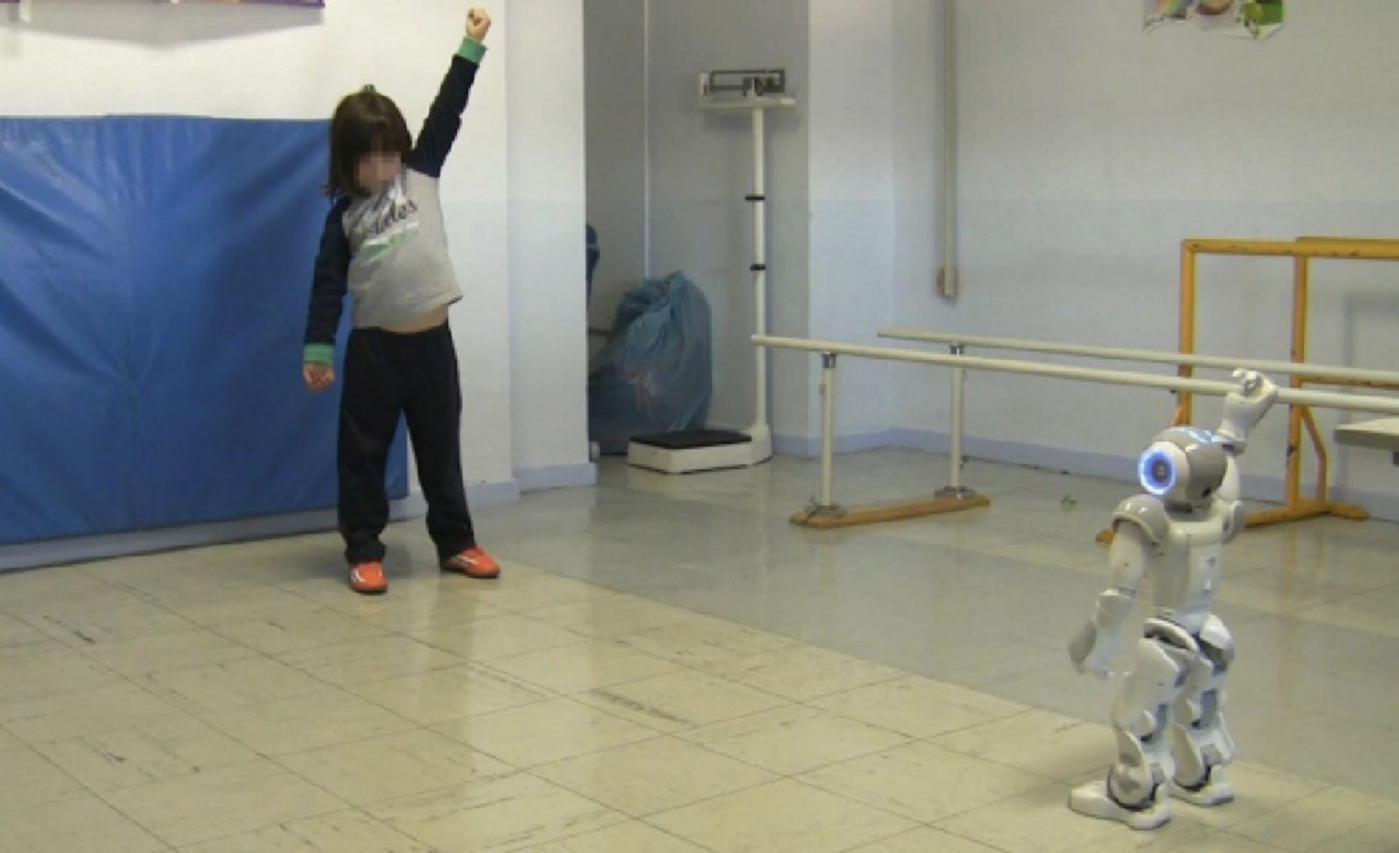
OBPP patient performing imitation game with NAO robot during trial at VRUH, Sevilla, Spain [16]

Motor rehabilitation with social interaction combining upper-limb training with playful cues, praise, and interactive gestures to strengthen musculature and dexterity [13, 58]. Robots guided repetitive movements in hospital and home contexts, sustaining motivation through adaptive feedback [48, 52, 59]. Longitudinal deployments reported modest but measurable improvements on standardized motor scales (MACS, QUEST, Mallet), with participants and caregivers rating robot-mediated sessions as more engaging than traditional therapy [51, 60] (Fig. 2).

Collectively, these studies show that socially assistive robots can address multiple therapeutic domains simultaneously, enhancing engagement while supporting speech, cognition, communication, play, and motor rehabilitation.

### Robot types and roles

#### Robot types

**Fig. 3:**
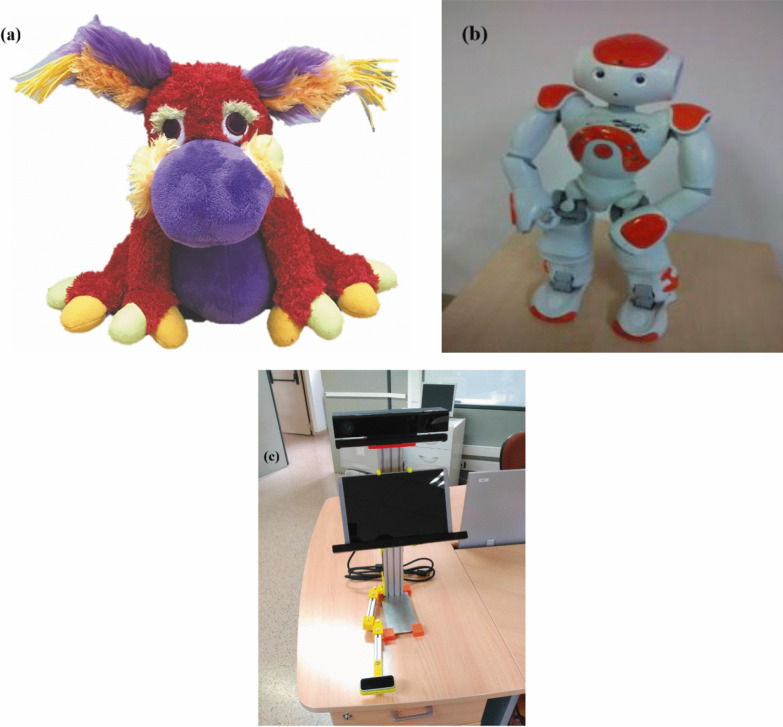
Examples of SARs in rehabilitation. **a** Animaloid SAR [61]. **b** Humanoid social robot NAO [57]. **c** Non-anthropomorphic platform [59]

The included studies deployed humanoid, animaloid, and non-anthropomorphic robots, with humanoids dominating the evidence base. Table 3 provides an overview of the robot platforms used across the included studies and highlights three main categories of socially assistive robots: humanoid, animaloid, and non-anthropomorphic designs. Examples of the different types of socially assistive robots are presented in Fig. 3. Humanoid robots were most common, particularly the NAO platform, used across multiple studies [41, 43–45, 48, 51–54, 62]. Standing 58 cm tall with 25 degrees of freedom (DOF) and multiple sensors, NAO enabled speech, vision, and tactile interaction [44]. It frequently acted as a social coach, exercise demonstrator, or conversational partner, supporting motor practice, communication, and social engagement in both clinics and home-based settings [45, 51, 52, 59, 60]. Other humanoids included a virtual robot in CoppeliaSim with articulated arms, hands, and facial expressions for gesture-based therapy [13], and a robot with 5 DOFs and an RGB-D sensor to track patient movement and provide visual feedback [49]. Animaloid robots appeared less frequently. Study [58] used a robotic stuffed animal with a soft, appealing design to encourage engagement in children with severe impairments. While not present in other included trials, broader CP rehabilitation literature cites platforms like PARO and PLEO [50], suggesting animaloid companions are a relevant but underexplored modality. Non-anthropomorphic robots prioritized function over appearance. Study [56] described a mobile, multisensory robot designed for home use, emphasizing adaptability and sensor integration. In [57], an adapted LEGO rover acted as a mobile play partner, engaging children in collaborative play rather than delivering structured therapy. Overall, humanoid robots—especially NAO—dominated due to their expressive, interactive, and adaptable design.

**Table 3:** Summary of social and rehabilitation robots in pediatric cerebral palsy interventions

Study	Robot type	Robot name	Adaptivity	Autonomy	Verbal cues used	Nonverbal cues used	Gamification used
Clark et al. [58]	Animaloid	Animated robotic stuffed animal	Limited—therapist-controlled	Semi-autonomous (“wizard mode”)	No	Movement, sound cues (chatter, chirps)	No
Peramalaiah et al. [61]	Non-anthropomorphic	Robotic Manipulandum Device (RMD)	None	Low autonomy	No	Movement-based input via handles	Yes (linked to commercial games)
Lu et al. [13]	Humanoid (virtual)	CoppeliaSim humanoid (14 DOF arms, 10 DOF hands)	Yes—real-time praise/feedback	Semi-autonomous	Questions, praise	Gestures (shrug, wave, thumbs-up)	Movement-based scenarios
Malik et al. [63]	Humanoid	NAO humanoid (25 DOF)	None	Low autonomy	Instructions, motivational cues	Body movement demos	GMFM motor-task scenarios
Gómez-Donoso et al. [56]	Non-anthropomorphic	Mobile multisensory robotic platform	Limited—adjusts parameters	Semi-autonomous	No	Movement patterns, sounds	Symbolic play, gesture integration into gameplay
González et al. [52]	Humanoid	NAO humanoid	High—real-time session adaptation	High autonomy	Yes	Yes	Yes
Malik et al. [53]	Humanoid	NAO humanoid	Limited	Semi-autonomous	Verbal prompts, feedback	Gesture demonstrations	Yes
Tabb et al. [48]	Humanoid	NAO humanoid	Limited	Semi-autonomous	Praise, suggestions	Head movements, posture changes	Scoring, progress indicators
Haze et al. [44]	Humanoid	NAO with ASR system	Improved speech recognition	–	Yes	–	–
Rahman et al. [54]	Humanoid	NAO humanoid	None	Low autonomy	Yes	Yes	Yes
Carrillo et al. [59]	Humanoid	NAO humanoid	None	High autonomy	Motivational statements, reminders	Gestures, LED prompts, tactile buttons	Yes
Pulido et al. [49]	Humanoid	NAOTherapist humanoid	Limited	Semi-autonomous	Verbal prompts	Gestures, animations	Interactive bilateral arm games
Pulido et al. [60]	Humanoid	NAOTherapist robot	Limited	Semi-autonomous	Coaching, feedback	Mirror/Simon movement demos	Mirror, Simon games
Gnjatović et al. [46]	Humanoid	MARKO humanoid	None	Low autonomy	Conversational prompts	–	–
Robles-Bykbaev et al. [45]	Humanoid	SPELTRA humanoid assistant	None	Low autonomy	Speech therapy prompts	–	Turn-taking games
Rios-Rincon et al. [57]	Non-anthropomorphic	Adapted LEGO rover	None	Low autonomy	–	Movement, lights, sounds	Play-based free interaction
Dennler et al. [51]	Humanoid	NAO humanoid	Adaptive feedback model	Semi-autonomous	Social feedback (encourage/reward)	Gesture recognition	Non-verbal communication game
Gong and Wu [47]	Non-anthropomorphic	HCI rehab system	Potential (speech/gesture recognition)	Concept only	Yes	Yes	Yes
Pirborj et al. [55]	Humanoid	Pepper humanoid	Limited (role-reversal)	Semi-autonomous	Yes	Yes	Sensory role-play
Lins et al. [50]	Animaloid	Various (PARO, PLEO, SPELTRA, Shelbytron, GoBot)	Not specified	Not specified	Possible	Possible	Possible

#### Verbal communication

Interaction relied on verbal, nonverbal, and gamified strategies, often combined for maximum engagement. Verbal communication included greetings, prompts, reminders, and praise [13, 45, 48–54, 60, 61, 63]. Some interventions used scripted motivational statements [59], while others offered follow-up questions [31] or contextual dialogue [13]. Advanced features included ASR tailored for dysarthric speech [24, 25], dialogue corpora to support turn-taking [46], and speech therapy games targeting articulatory and semantic accuracy [45, 60].

#### Non-verbal strategies

Nonverbal strategies supported or substituted speech through gestures, LED signals, touch responses, or posture demonstrations. Examples include chirping and locomotion [58], full-body task demonstrations [63], expressive gestures [3, 27], and movement cues paired with verbal feedback [48, 49, 59]. In [51], children communicated nonverbally with the robot, which responded with encouragement.

#### Gamification

Gamification was commonly applied [13, 30, 47, 48, 52–54, 57, 61], embedding therapy into enjoyable activities like mirror and Simon games, memory challenges, role-play, and scoring systems. Robots demonstrated therapy tasks [3, 40] linked physical movements to game controls [52–54], or used progress tracking and rewards to sustain adherence [48, 59]. A LEGO rover supported embodied free play [57], while multimodal designs enabled both speech- and gesture-based control [47].

#### Degree of autonomy and adaptivity

##### High autonomy robots

Details of robot adaptivity, autonomy, and features across studies are summarized in Table 3. Robots ranged from low-autonomy, therapist-dependent systems to semi- and high-autonomy platforms with adaptive features. High-autonomy examples were rare, including systems that autonomously scheduled or adapted therapy [52] or delivered scripted routines with minimal supervision [59], though progress tracking and personalization still require caregiver input.

##### Semi-autonomous robots

Semi-autonomous robots formed the largest group, typically running pre-programmed routines or games with adjustments made via sensors or therapist input. Examples include daily therapist-programmed exercises [44], Kinect-based motion tracking [13], adaptive multisensory platforms [56], and structured routines delivered by NAO [45, 46, 49, 53, 60], usually under therapist supervision.

##### Low-autonomy robots

Low-autonomy systems, common in clinical and educational settings, relied on static scripts [54, 63], fixed mechanical assistance [61], or full adult mediation, as in rover-based play [57]. Even when engagement-based feedback was incorporated [51], decision-making remained limited. More recent approaches seek to advance autonomy, such as role-reversal scenarios, positioning the robot as a tutee [55], integrated speech and gesture recognition [47], and ASR tailored for dysarthric speech [44], though these remain at the pilot stage.

### Intervention settings and protocols

Intervention settings were described in terms of location, frequency/duration, and delivery mode (therapist-led vs. autonomous). Most studies (n = 9) were conducted in rehabilitation clinics, reflecting access to equipment, supervision, and controlled conditions [3, 27, 30, 35, 36, 40, 41, 58, 61, 63]. Mixed settings (n = 2) combined clinic, home, and school contexts to extend practice opportunities [56, 58]. Purely home-based trials (n = 2) explored autonomous or parent-supported therapy [13, 31], while two studies were in controlled research rooms [32, 63]. University rehabilitation facilities (n = 2) offered both treatment and experimental control [2, 28]. Several reports did not specify the setting. Overall, clinics dominated due to patient availability and structured environments.

In terms of frequency, duration, and intensity, it can be seen that protocols varied widely, from a single one-hour visits [52] to multi-week regimens. Short-term designs included 4–5 sessions of 10–60 min [56, 63]. Longer programs lasted 8–12 weeks with 2–3 sessions per week [48, 60, 61], each lasting 20–45 min. Intensive interventions were also described: e.g., 30 sessions of 25–30 min [52], or 73 speech-therapy sessions supplemented by app-based home practice [45]. Play-based designs used multiple-baseline protocols with 10–15-min sessions [57]. Overall, most interventions scheduled 1–3 sessions weekly for 20–40 min, often embedded in broader therapy routines.

#### Levels of autonomy in interventions

Regarding autonomy, most interventions were therapist-led (n = 10) or mixed-mode (n = 9), while only a small number of studies reported fully autonomous or unspecified modes (n = 2). Most studies were therapist-led or mixed. In fully therapist-led interventions, robots operated via pre-programmed scripts or manual control with no adaptive capacity [44, 46, 54, 56–58, 61, 63]. Mixed approaches were common: therapists set goals and parameters, while robots autonomously guided exercises, delivered feedback, or supported home practice [13, 45, 48, 49, 51–53, 59, 60]. For example, NAOTherapist [60] allowed clinicians to personalize plans, after which the robot ran exercises autonomously. Similarly, [44] enabled therapist-programmed home sessions executed by the robot without supervision. In [52, 53], robots autonomously scheduled or executed therapy once goals were defined. Some systems provided automated feedback within a researcher-controlled protocol [51]. Fully autonomous sessions remained rare, with most platforms relying on therapist setup, monitoring, or troubleshooting.

In summary, interventions were predominantly delivered in clinical rehabilitation settings with structured but heterogeneous protocols. Sessions typically last 20–40 min, 1–3 times per week, with delivery modes ranging from therapist-dependent to semi-autonomous execution. Fully autonomous therapy remains uncommon, highlighting the continuing need for therapist involvement in pediatric CP rehabilitation with social robots.

### Reported outcome

#### Improvements in speech or language scores

Several studies reported improvements in speech and language following robot-assisted therapy. Speech and language outcomes across reviewed studies are summarized in Table 3. A large trial with 29 children and 73 sessions documented gains in phonological production, morphosyntax, and semantics, with rapid adaptation and comparable outcomes to therapist-led sessions [45]. Technical advances were also noted: one study demonstrated improved automatic speech recognition for dysarthric speech through reduced error rates [44], while another achieved ≥ 96% gesture and voice recognition accuracy, confirming the reliability of automated assessment, though without clinical endpoints [47, 56]. Overall, outcomes ranged from measurable linguistic progress to technical performance improvements, underscoring both therapeutic potential and the growing robustness of assistive systems. Table 4 summarizes the studies reporting speech and language outcomes, including robot platform, participants, intervention focus, and key outcomes.

**Table 4:** Summary of speech and language outcome studies

Study	Robot platform	Participants	Intervention focus	Key outcomes reported
[49]	Not specified	Not specified	Dysarthric speech recognition	Reduced CER and WER in ASR
[50]	Robotic assistant	29 children, 73 sessions	Phonological production, morphosyntax, semantics	Positive gains across all three areas
[52]	HCI training system	Not specified	Action/voice recognition modules	≥ 96% recognition accuracy

Overall, robot-assisted interventions reported promising gains in phonological production, morphosyntax, and semantics in small-scale studies (e.g., the 29-child trial [50]). Some studies focused on technical validation (improved ASR accuracy for dysarthric speech [49]) rather than clinical endpoints. The evidence base remains limited by small sample sizes and heterogeneous outcomes, so results should be interpreted cautiously.

#### Cognitive engagement metrics

Cognitive engagement outcomes consistently indicated that socially assistive robots sustain attention, improve coordination, and enhance task adherence. Across the included studies, cognitive engagement was measured using observational methods and quantitative timing metrics (each n = 2), followed by therapist rating scales (n = 1) and technology-acceptance questionnaires (n = 1). In addition, summary of cognitive engagement measurement studies is given in Table 4. Motor-cognitive metrics showed significant improvements, such as reduced Movement Completion Time in one participant (p = 0.007) [13]. Observational measures reported enhanced concentration and eye-hand coordination during gamified tasks [61]. Technical metrics also reflected system efficiency, with heuristic planning algorithms reducing session setup times [52]. Engagement was further validated by high therapist ratings (≥ 60% across motivation and clarity domains) [63] and reliable observational coding (κ = 0.73) [51]. Children and therapists expressed a clear preference for embodied robots over screen-based agents, with higher ratings for enjoyment (p = 0.018) and companionship (p = 0.026) [51]. Extended deployments confirmed stable acceptance and high satisfaction, with patient ratings exceeding 4.6/5 and caregiver/clinician scores averaging ~ 4.3–4.5/5 [60]. Table 5 summarizes measurement approaches for cognitive engagement (measurement type, metrics, key findings, and statistical significance).

**Table 5:** Summary of cognitive engagement measurement studies

Study	Measurement type	Specific metrics	Key findings	Statistical significance
[61]	Parent-reported cognitive improvements	Concentration, eye-hand coordination	Improved concentration and coordination	Not specified
[13]	Motor-cognitive timing	MRT, MCT	MCT improvement for Patient A	p = 0.007
[63]	Therapist ratings	Multiple engagement dimensions	> 60% scores across most categories	Not specified
[52]	Planning efficiency	Session planning times	Heuristic vs. blind selection comparison	Time reductions reported
[16]	Technology acceptance	TAM questionnaire scores	High satisfaction and utility ratings	Not specified
[20]	Binary engagement analysis	Engagement states, preference measures	Robot preference over screen agent	p = 0.018 (Enjoyment), p = 0.026 (Companionship)

The studies used diverse engagement measures (observational coding, timing metrics, therapist ratings, TAM questionnaires). Where statistical tests were reported, some motor-cognitive metrics reached significance (e.g., MCT improvement, p = 0.007 [12]); however, heterogeneity in metrics limits cross-study aggregation and points to a need for standardized engagement measures.

#### Parent and clinician feedback

Parents and clinicians consistently report positive experiences with robotic interventions. Information on parents and clinicians' feedback across studies is summarized in Table 5. Parents highlighted increased motivation, functional gains, and engagement in therapy [59, 61]. Therapists emphasized reduced workload, reliable demonstrations, and greater ability to maintain patient focus [13, 59]. Limitations included battery life and sensitivity to patient mood [59]. Quantitative data supported these findings: during a 2-month deployment, caregivers rated utility and ease of use at ~ 4.5/5, while clinicians gave ratings around 4.3/5 for perceived empathy and usefulness [60]. Parents often describe robots as effective motivators, reinforcing their therapeutic potential [58, 59]. Table 6 summarizes parent and clinician feedback studies, listing respondent type, assessment method, key feedback areas, and overall response.

**Table 6:** Summary of parent and clinician feedback studies

Study	Respondent type	Assessment method	Key feedback areas	Overall response
[61]	Parents	Qualitative interviews	Acceptance, motivation, functional improvements	High acceptance
[13]	Therapists	Clinical observations	Engagement, manual intervention, and workload	Reduced workload, increased engagement
[59]	Therapists and parents	Early feedback	Exercise demonstration, motivation, limitations	Useful and engaging, with some technical limitations
[16]	Relatives, caregivers, clinical experts	TAM questionnaires	Utility, ease-of-use, empathy/interaction	High satisfaction (4.33–4.5/5)

Parents and clinicians generally reported high acceptance and perceived benefits (motivation, reduced workload, clearer demonstrations). Most feedback is qualitative or from short deployments, so although responses are positive, longer-term and quantitative acceptability studies are needed to confirm sustained utility.

#### Child acceptance and engagement levels

Children demonstrated strong acceptance and active participation across interventions. Across studies, child acceptance and engagement were high: 75% of studies reported high acceptance, 17% moderate acceptance, and 8% mixed responses. In one trial, 5 of 8 children showed higher engagement with robots compared to switch-adapted toys, measured via behaviors such as visual regard, vocalization, and affect [58]. Anecdotal evidence included children expressing social attachment to robots (e.g., asking “Are we friends?”) [58]. Gamified designs were particularly effective, eliciting enthusiasm and sustained participation [61]. Clinicians reported increased attempts and activity during robot-led sessions, with even severely impaired children showing incremental progress [13, 53, 54].

Quantitative data confirmed these patterns. Robot-mediated play interventions improved Test of Playfulness scores with moderate-to-large effect sizes (IRD = 0.58–1.00), and parent-rated occupational performance and satisfaction improved for all participants, persisting at follow-up [57]. Intensive programs, such as a 10-day HABIT camp with the NAOTherapist, demonstrated high usability, strong social acceptance, and improved adherence to rehabilitation tasks [49]. Extended use fostered positive emotional bonds, with children maintaining focus and motivation throughout [49, 59].

Across all the studies, children, parents, and clinicians consistently reported positive outcomes, including improvement in speech/language skills, higher engagement, and strong satisfaction. While some studies prioritized technical validation, the convergence of therapeutic progress, engagement, and stakeholder support underscores the feasibility of integrating socially assistive robotics into pediatric CP rehabilitation. Further large-scale trials are needed to assess the durability of effects and long-term outcomes.

## Discussion

A major limitation across studies was small sample size, with most including fewer than 20 participants. While this restricts statistical power and generalizability, small-scale feasibility trials are valuable in pediatric populations, as they enable iterative refinement of robotic platforms, identification of usability issues, and close monitoring of safety and engagement [59]. Short-term studies similarly allowed researchers to test novel engagement strategies, adjust difficulty levels, and gather rapid feedback from therapists and participants [51, 58, 63, 64]. However, few studies extended interventions over multiple weeks or conducted follow-up evaluations, leaving limited evidence on durability, transfer of skills beyond the therapy context, or dose–response relationships [49, 57, 60]. Larger, longitudinal trials will be essential to establish efficacy and sustainability.

Heterogeneity in outcome measures further complicates cross-study comparisons. Clinical endpoints included standardized scales for motor skills (MACS, Mallet, QUEST), play and participation measures (ToP, COPM), and various speech-language assessments [45, 57, 60]. Engagement was quantified using behavioral coding, task logs, therapist/parent ratings, or usability scales such as SUS and TAM [51, 59, 60, 64]. Technical studies reported performance metrics such as speech recognition error rates or gesture recognition accuracy [44, 47], which provide valuable engineering insights but are difficult to relate directly to clinical outcomes. While this diversity reflects the multidimensional nature of SAR interventions, a lack of standardized reporting frameworks makes it challenging to synthesize evidence systematically.

Despite these methodological limitations, several key findings emerged. Robot-assisted interventions targeting speech and language demonstrated improvements in phonological production, morphosyntax, and semantics, with technical advances such as dysarthric speech recognition offering new therapeutic possibilities [44, 45, 47]. Cognitive and social-pragmatic outcomes were also addressed, with SARs supporting attention, memory, turn-taking, and symbolic play through gamified tasks and role-playing scenarios [44, 48, 54, 55, 57, 63]. These findings highlight the potential of SARs as multimodal platforms capable of integrating cognitive, linguistic, and socio-emotional goals within engaging therapeutic contexts.

Across studies, children consistently demonstrated strong acceptance of SARs, often perceiving them as companions or play partners [58, 61]. Engagement indicators—including eye contact, vocalization, and active participation—were sustained over repeated sessions, with gamification and physical embodiment emerging as key factors for motivation [49, 52, 57, 61]. Parents and clinicians echoed these positive evaluations, reporting increased therapy adherence, improved functional outcomes, and reduced therapist workload [13, 59, 60]. The predominance of humanoid platforms, particularly NAO [45, 46, 49, 51–53, 59, 60], demonstrates the utility of sensor-rich, conversational designs, but also underscores the need for broader platform diversity to test scalability and generalizability across contexts.

Notably, children with severe CP and multilingual backgrounds remain underrepresented. Only a small number of studies included participants at GMFCS/MACS levels IV-V, despite evidence that SARs may be particularly beneficial for maintaining engagement in children with high support needs [57, 58]. Similarly, most speech-language interventions were single-language, with little exploration of cross-lingual adaptation or multilingual deployment [45, 46]. Technical progress in dysarthric and multilingual ASR [44] has yet to be integrated into clinical trials, leaving gaps in understanding how SARs can support linguistically diverse populations. These gaps highlight an urgent need for SAR systems designed with accessibility in mind, including multilingual interaction capabilities and interfaces tailored for children with profound motor and communication impairments.

From a clinical perspective, SARs are best understood as complements rather than replacements for therapists. By automating repetitive demonstrations, facilitating structured practice, and maintaining engagement, SARs can free clinicians to focus on more complex, individualized interventions [13, 59]. This is especially relevant in underserved or remote regions, where access to qualified speech-language pathologists (SLPs) is limited. SARs could extend therapeutic reach through hybrid clinic–home models, remote monitoring, and consistent therapy delivery. However, challenges remain in terms of cost, technical infrastructure, and the need for professional training. Addressing these barriers will be crucial for the equitable integration of SARs into rehabilitation practice.

Taken together, the evidence suggests that SARs hold considerable promise for enhancing cognitive, speech, and communication rehabilitation in children with CP, but progress toward clinical translation will require larger, standardized, and longitudinal studies. Establishing common outcome frameworks, including both clinical and engagement metrics, will be critical for generating robust evidence. In parallel, attention to real-world implementation—encompassing issues such as autonomy, workflow integration, and accessibility for individuals with severe and multilingual needs—will determine whether SARs can evolve from exploratory prototypes into sustainable, widely adopted tools in pediatric rehabilitation.

## Limitations and future directions

This scoping review has several limitations. Firstly, the search strategy was restricted to peer-reviewed studies in English, which may exclude relevant studies published in other languages. Non-peer-reviewed literature such as conference abstracts and preprints, as well as unindexed proceedings, have not been included, and may contain valuable evidence. Additionally, traditional databases were utilized, which means early-stage and experimental research indexed primarily in OpenAlex and Dimensions may not have been considered.

All the studies included have small sample sizes, and significant heterogeneity in methodology, including intervention duration, robotic platform, and outcome measures, limits the comparability of the cross-sectional results. Furthermore, children with severe cerebral palsy (Gross Motor Function Classification System/Manual Assessment of Children's Skills IV–V) and multilingual participants were underrepresented, reducing the generalizability of the findings.

Future reviews should consider adopting a real-time review model in order to accommodate new evidence as it becomes available over time. Including gray literature and using broader index-based repositories could contribute to a more comprehensive synthesis of the rapidly expanding field of social robotics.

## Conclusion

This scoping review systematically charted the emerging landscape of SARs in cognitive, speech, and communication rehabilitation for children with cerebral palsy. Across diverse studies, SARs demonstrated consistent benefits in speech production, symbolic play, attention, and engagement. Children often showed greater motivation and willingness to participate in therapy, while parents and clinicians reported high acceptance and perceived functional gains. These outcomes were observed in various contexts—including clinics, schools, and home-based programs—using humanoid and animaloid platforms, underscoring the flexibility of SAR deployment. Collectively, the evidence positions SARs as promising adjunctive tools within multidisciplinary rehabilitation frameworks.

These limitations are small samples, short intervention periods, heterogeneous designs, and limited longitudinal data-make cross-study comparisons difficult and prevent firm conclusions about long-term clinical efficacy. Nonetheless, SARs consistently demonstrated feasibility, acceptability, and therapeutic potential, providing an important foundation for the next phase of clinical research.

Looking forward, SARs hold distinct advantages as tools for rehabilitation: they offer stimulating, socially rich environments, support repetitive practice, and enable personalization that sustains children’s motivation—key drivers of therapeutic success in CP. They can extend therapist reach, reduce workload, and provide consistent demonstrations across sessions, thereby complementing rather than replacing conventional therapy.

Future research should move beyond feasibility studies toward larger, well-powered, and multi-week trials with standardized outcome frameworks. These should integrate validated clinical measures of motor function and participation alongside harmonized engagement and usability metrics. Importantly, children across the full GMFCS/MACS spectrum, including those with severe impairments, should be represented to ensure generalizability. Advances in speech technologies—particularly for dysarthric and multilingual populations—need systematic evaluation within therapy protocols. Clinical studies should also assess levels of robot autonomy, personalization, and workflow integration, alongside practical endpoints such as safety, setup time, and co-design responsiveness to patient mood and context.

Ultimately, the integration of SARs into pediatric rehabilitation will depend on clear usage guidelines, attention to real-world implementation, and hybrid clinic–home models supported by remote monitoring and data sharing. If pursued systematically, SARs can evolve from experimental prototypes into clinically validated tools that enrich rehabilitation experiences, improve therapy adherence, and enhance the cognitive and communicative development of children with cerebral palsy.

## Data Availability

The data extracted and analyzed during this review are available from the corresponding author on reasonable request.

## Abbreviations

CP: Cerebral palsy
SAR / SARs: Socially assistive robot(s)
GMFCS: Gross motor function classification system
MACS: Manual ability classification system
CFCS: Communication function classification system
PRISMA-ScR: Preferred reporting items for systematic reviews and meta-analyses extension for scoping reviews
ASR: Automatic speech recognition
TAM: Technology acceptance model
HCI: Human–computer interaction
DOF: Degrees of freedom
CER: Character error rate
WER: Word error rate
ToP: Test of playfulness
COPM: Canadian occupational performance measure
QUEST: Quality of upper extremity skills test
OBPP: Obstetric brachial plexus palsy
RMD: Robotic manipulandum device
MRT: Movement reaction time
MCT: Movement completion time

## References

[1] Schiariti V, et al., Implementation of the international classification of functioning, disability, and health (ICF) core sets for children and youth with cerebral palsy: global initiatives promoting optimal functioning. Int J Environ Res Publ Health. 2018;15(9):1899 [Online]. Available: https://www.mdpi.com/1660-4601/15/9/1899. 10.3390/ijerph15091899 PMC6163506 30200412

[2] Stavsky M, Mor O, Mastrolia SA, Greenbaum S, Than NG, Erez O. Cerebral palsy—trends in epidemiology and recent development in prenatal mechanisms of disease, treatment, and prevention. Front Pediatr Rev. 2017;5 (in English). 10.3389/fped.2017.00021. 10.3389/fped.2017.00021 PMC5304407 28243583

[3] Katangwe TJ, et al. Variables included in cerebral palsy registries globally: a scoping review. Dev Med Child Neurol. 2024;66(9):1148–56. 10.1111/dmcn.15908. 38530807 10.1111/dmcn.15908 PMC11579827

[4] Malik NA, Hanapiah FA, Rahman RAA, Yussof H. Emergence of socially assistive robotics in rehabilitation for children with cerebral palsy: a review. Int J Adv Robot Syst. 2016;13(3):135. 10.5772/64163.

[5] Arias Valdivia JT, Gatica Rojas V, Astudillo CA. Deep learning-based classification of hemiplegia and diplegia in cerebral palsy using postural control analysis. Sci Rep. 2025;15(1):8811. 10.1038/s41598-025-93166-3. 40087338 10.1038/s41598-025-93166-3 PMC11909225

[6] Fluss J, Lidzba K. Cognitive and academic profiles in children with cerebral palsy: a narrative review. Ann Phys Rehabil Med. 2020;63(5):447–56. 32087307 10.1016/j.rehab.2020.01.005

[7] Hansen JE, Støve LL, Væver MS, Røhder K. Socio-emotional development in young children with cerebral palsy: a scoping review. Child Care Health Dev. 2025;51(4):e70130. 40605235 10.1111/cch.70130 PMC12223170

[8] Salomon I. Neurobiological insights into cerebral palsy: a review of the mechanisms and therapeutic strategies. Brain Behav. 2024;14(10):e70065. 39378294 10.1002/brb3.70065 PMC11460637

[9] Uhre CF, Caspersen ID, Lose C, Rackauskaite G, Robotham R, Hoei-Hansen CE. Cognitive functioning in children and adolescents with cerebral palsy: protocol for the Danish CPCog-Youth study. BMC Pediatr. 2024;24(1):836. 39725891 10.1186/s12887-024-05305-w PMC11673341

[10] Zaghloul N, Ahmed M. Pathophysiology of periventricular leukomalacia: what we learned from animal models. Neural Regen Res. 2017;12(11):1795–6. 29239318 10.4103/1673-5374.219034 PMC5745826

[11] Dogan S, Colak A. Social robots in the instruction of social skills in autism: a comprehensive descriptive analysis of single-case experimental designs. Disabil Rehabil Assist Technol. 2024;19(2):325–44. 10.1080/17483107.2022.2087772. 35758001 10.1080/17483107.2022.2087772

[12] Buitrago JA, Bolaños AM, Caicedo Bravo E. A motor learning therapeutic intervention for a child with cerebral palsy through a social assistive robot. Disabil Rehabil Assist Technol. 2020;15(3):357–62. 30806105 10.1080/17483107.2019.1578999

[13] Lu Z, et al. Development of a virtual robot rehabilitation training system for children with cerebral palsy: an observational study. Sensors. 2024;24(24):8138 [Online]. Available: https://www.mdpi.com/1424-8220/24/24/8138. 10.3390/s24248138 PMC11679212 39771873

[14] Gardeazabal X, Abascal J. Use of robots for play by children with cerebral palsy. Proceedings. 2019;31(1):75 [Online]. Available: https://www.mdpi.com/2504-3900/31/1/75.

[15] Ferm UM, Claesson BK, Ottesjö C, Ericsson S. Participation and enjoyment in play with a robot between children with cerebral palsy who use AAC and their peers. Augment Altern Commun. 2015;31(2):108–23. 10.3109/07434618.2015.1029141. 25921358 10.3109/07434618.2015.1029141

[16] Torring MF, Logacjov A, Braendvik SM, Ustad A, Roeleveld K, Bardal EM. Validation of two novel human activity recognition models for typically developing children and children with Cerebral Palsy. PLoS ONE. 2024;19(9):e0308853. 10.1371/journal.pone.0308853. 39312531 10.1371/journal.pone.0308853 PMC11419372

[17] Rausch R, et al. The mental health of children with cerebral palsy: a review of the last five years of research. J Clin Med. 2025. 10.3390/jcm14124364. 40566110 10.3390/jcm14124364 PMC12194703

[18] Shank LK, Kaufman J, Leffard S, Warschausky S. Inspection time and attention-deficit/hyperactivity disorder symptoms in children with cerebral palsy. Rehabil Psychol. 2010;55(2):188. 20496973 10.1037/a0019601 PMC2877285

[19] Pirila S, van der Meere JJ, Rantanen K, Jokiluoma M, Eriksson K. Executive functions in youth with spastic cerebral palsy. J Child Neurol. 2011;26(7):817–21. 21398561 10.1177/0883073810392584

[20] Dogruoz Karatekin B, Icagasioglu A. Quality of life, participation, and functional status in cerebral palsy: a 13-year follow-up study. Medeniyet Med J. 2022;37(1):105–12. 10.4274/MMJ.galenos.2022.54920. 10.4274/MMJ.galenos.2022.54920 PMC8939458 35306797

[21] Korkalainen J, McCabe P, Smidt A, Morgan C. Motor speech interventions for children with cerebral palsy: a systematic review. J Speech Lang Hear Res. 2023;66(1):110–25. 36623233 10.1044/2022_JSLHR-22-00375

[22] Hoang KC, Pham VM. Efficacy of the comprehensive intervention model for children < 6 years of age with spastic cerebral palsy. World Acad Sci J. 2025;7(3):48.

[23] Stadskleiv K, Lorentzen LE, Hollung SJ. Cognitive assessment practices of children with cerebral palsy: a national cohort study. Dev Neurorehabil. 2025. 10.1080/17518423.2025.2526357. 40621621 10.1080/17518423.2025.2526357

[24] García-Galant M, et al. Understanding social cognition in children with cerebral palsy: exploring the relationship with executive functions and the intervention outcomes in a randomized controlled trial. Eur J Pediatr. 2024;183(9):3997–4008. 38951253 10.1007/s00431-024-05635-y PMC11322257

[25] Hilderley AJ, Wright FV, Taylor MJ, Chen JL, Fehlings D. Functional neuroplasticity and motor skill change following gross motor interventions for children with diplegic cerebral palsy. Neurorehabil Neural Repair. 2023;37(1):16–26. 36524254 10.1177/15459683221143503 PMC9896542

[26] Lindsay S. Child and youth experiences and perspectives of cerebral palsy: a qualitative systematic review. Child Care Health Dev. 2016;42(2):153–75. 26754030 10.1111/cch.12309

[27] Breazeal C, Dautenhahn K, Kanda T. Social robotics. In: Springer handbook of robotics; 2016. p. 1935–72.

[28] Ragno L, Borboni A, Vannetti F, Amici C, Cusano N. Application of social robots in healthcare: review on characteristics, requirements, technical solutions. Sensors. 2023;23(15):6820. 37571603 10.3390/s23156820 PMC10422563

[29] Cifuentes CA, Veneman JF, Rocon E, Rodriguez-Guerrero C. Interfacing humans and machines for rehabilitation and assistive devices. 2022;8:796431. 10.3389/frobt.2021.796431 PMC8762109 35047569

[30] Rasouli S, Gupta G, Nilsen E, Dautenhahn K. Potential applications of social robots in robot-assisted interventions for social anxiety. Int J Soc Robot. 2022;14(5):1–32. 35096198 10.1007/s12369-021-00851-0 PMC8787185

[31] Kabacińska K, Prescott TJ, Robillard JM. Socially assistive robots as mental health interventions for children: a scoping review. Int J Soc Robot. 2021;13(5):919–35.

[32] Taheri A, Alemi M, Meghdari A, Pouretemad H, Holderread S. Clinical application of humanoid robots in playing imitation games for autistic children in Iran. Procedia Soc Behav Sci. 2015;176:898–906.

[33] Figliano G, Manzi F, Tacci AL, Marchetti A, Massaro D. Ageing society and the challenge for social robotics: a systematic review of socially assistive robotics for MCI patients. PLoS ONE. 2023;18(11):e0293324. 10.1371/journal.pone.0293324. 38033146 10.1371/journal.pone.0293324 PMC10688856

[34] Cano S, González CS, Gil-Iranzo RM, Albiol-Pérez S. Affective communication for socially assistive robots (sars) for children with autism spectrum disorder: a systematic review. Sensors. 2021;21(15):5166. 34372402 10.3390/s21155166 PMC8347754

[35] Llamas-Ramos R, Sánchez-González JL, Llamas-Ramos I. Robotic systems for the physiotherapy treatment of children with cerebral palsy: a systematic review. Int J Environ Res Public Health. 2022;19(9):5116. 35564511 10.3390/ijerph19095116 PMC9100658

[36] Fahr A, Keller JW, Van Hedel HJ. A systematic review of training methods that may improve selective voluntary motor control in children with spastic cerebral palsy. Front Neurol. 2020;11:572038. 33343485 10.3389/fneur.2020.572038 PMC7746811

[37] Bonanno M, et al. Rehabilitation of gait and balance in cerebral palsy: a scoping review on the use of robotics with biomechanical implications. J Clin Med. 2023;12(9):3278. 37176718 10.3390/jcm12093278 PMC10179520

[38] Lewis TT, Kim H, Darcy-Mahoney A, Waldron M, Lee WH, Park CH. Robotic uses in pediatric care: a comprehensive review. J Pediatr Nurs. 2021;58:65–75. 33360676 10.1016/j.pedn.2020.10.016

[39] Zanatta F, Giardini A, Pierobon A, D’Addario M, Steca P. A systematic review on the usability of robotic and virtual reality devices in neuromotor rehabilitation: patients’ and healthcare professionals’ perspective. BMC Health Serv Res. 2022;22(1):523. 35443710 10.1186/s12913-022-07821-w PMC9020115

[40] Blankenship MM, Bodine C. Socially assistive robots for children with cerebral palsy: a meta-analysis. IEEE Trans Med Robot Bion. 2020;3(1):21–30.

[41] Dawe J, Sutherland C, Barco A, Broadbent E. Can social robots help children in healthcare contexts? A scoping review. BMJ Paediatr Open. 2019;3(1):e000371. 30815587 10.1136/bmjpo-2018-000371 PMC6361370

[42] Komendziński T, Mikołajewska E, Mikołajewski D, Dreszer J, Bałaj B. Cognitive robots in the development and rehabilitation of children with developmental disorders. Bio-Algorithms Med-Syst. 2016;12(3):93–8.

[43] Yuan F, Klavon E, Liu Z, Lopez RP, Zhao X. A systematic review of robotic rehabilitation for cognitive training. Front Robot AI. 2021;8:605715. 34046433 10.3389/frobt.2021.605715 PMC8144708

[44] Haze K, Takashima R, Takiguchi T. Speech recognition for a person with cerebral palsy using whisper fine-tuned on Japanese and English dysarthric speech. In: 2024 IEEE 13th global conference on consumer electronics (GCCE). IEEE; 2024. p. 419–20.

[45] Robles-Bykbaev V, et al. Robotic assistant for support in speech therapy for children with cerebral palsy. In: 2016 IEEE international autumn meeting on power, electronics and computing (ROPEC). IEEE; 2016. p. 1–6.

[46] Gnjatović M, et al. Pilot corpus of child-robot interaction in therapeutic settings. In: 2017 8th IEEE international conference on cognitive infocommunications (CogInfoCom). IEEE; 2017. p. 000253–8.

[47] Gong C, Wu G. Design of cerebral palsy rehabilitation training system based on human-computer interaction. In: 2021 international wireless communications and mobile computing (IWCMC). IEEE; 2021. p. 621–5.

[48] Tabb D, et al. Rehabilitation engagement at home with a socially assistive robot for pediatric adherence (REHAB-PAL)—a feasibility study. Arch Phys Med Rehabil. 2025;106(4):e175–6.

[49] Pulido JC, et al. A gamified social robotics platform for intensive therapies in neurorehabilitation. Intell Serv Robot. 2024;17(3):419–43. 10.1007/s11370-024-00521-w.

[50] Lins AA, de Oliveira JM, Rodrigues JJ, de Albuquerque VHC. Robot-assisted therapy for rehabilitation of children with cerebral palsy-a complementary and alternative approach. Comput Human Behav. 2019;100:152–67.

[51] Dennler N, Yunis C, Realmuto J, Sanger T, Nikolaidis S, Matarić M. Personalizing user engagement dynamics in a non-verbal communication game for cerebral palsy. In: 2021 30th IEEE international conference on robot & human interactive communication (RO-MAN). IEEE; 2021. p. 873–9.

[52] González JC, Pulido JC, Fernández F. A three-layer planning architecture for the autonomous control of rehabilitation therapies based on social robots. Cogn Syst Res. 2017;43:232–49. 10.1016/j.cogsys.2016.09.003.

[53] Malik NA, Yussof H, Hanapiah FA, Rahman RAA, Basri HH. Human-robot interaction for children with cerebral palsy: reflection and suggestion for interactive scenario design. Procedia Comput Sci. 2015;76:388–93. 10.1016/j.procs.2015.12.315.

[54] Rahman RAA, Hanapiah FA, Basri HH, Malik NA, Yussof H. Use of humanoid robot in children with cerebral palsy: the ups and downs in clinical experience. Procedia Comput Sci. 2015;76:394–9. 10.1016/j.procs.2015.12.316.

[55] Pirborj LM, Alnajjar F, Shafigh S. Empowering helpers: reversing roles in paediatric rehab with humanoid robots and sensory games. In: 2024 international conference on intelligent environments (IE). IEEE; 2024. p. 105–8.

[56] Gomez-Donoso F, et al. A robotic platform for customized and interactive rehabilitation of persons with disabilities. Pattern Recognit Lett. 2017;99:105–13. 10.1016/j.patrec.2017.05.027.

[57] Ríos-Rincón AM, Adams K, Magill-Evans J, Cook A. Playfulness in children with limited motor abilities when using a robot. Phys Occup Ther Pediatr. 2016;36(3):232–46. 26566226 10.3109/01942638.2015.1076559

[58] Clark C, Sliker L, Sandstrum J, Burne B, Haggett V, Bodine C. Development and preliminary investigation of a semiautonomous Socially Assistive Robot (SAR) designed to elicit communication, motor skills, emotion, and visual regard (engagement) from young children with complex cerebral palsy: a pilot comparative trial. Adv Hum-Comput Interact. 2019;2019(1):2614060.

[59] Martí Carrillo F, et al. Adapting a general-purpose social robot for paediatric rehabilitation through in situ design. ACM Trans Hum Robot Interact. 2018;7(1):1–30.

[60] Pulido JC, et al. A socially assistive robotic platform for upper-limb rehabilitation: a longitudinal study with pediatric patients. IEEE Robot Autom Mag. 2019;26(2):24–39.

[61] Peramalaiah MK, et al. Evaluation of a game-based mechatronic device for rehabilitation of hand-arm function in children with cerebral palsy: feasibility randomized controlled trial. JMIR Rehabil assistive Technol. 2025;12(1):e65358. 10.2196/65358 PMC11888099 39964707

[62] Munn Z, et al. What are scoping reviews? Providing a formal definition of scoping reviews as a type of evidence synthesis. JBI Evid Synth. 2022;20(4):950–2. 35249995 10.11124/JBIES-21-00483

[63] Malik NA, Yussof H, Hanapiah FA. Interactive scenario development of robot-assisted therapy for cerebral palsy: a face validation survey. Procedia Comput Sci. 2017;105:322–7. 10.1016/j.procs.2017.01.229.

[64] Mahdi H, Akgun SA, Saleh S, Dautenhahn K. A survey on the design and evolution of social robots—past, present and future. Robot Auton Syst. 2022;156:104193.

